# Determinants of bone health in elderly Japanese men: study design and key findings of the Fujiwara-kyo Osteoporosis Risk in Men (FORMEN) cohort study

**DOI:** 10.1186/s12199-021-00972-y

**Published:** 2021-04-23

**Authors:** Yuki Fujita, Junko Tamaki, Katsuyasu Kouda, Akiko Yura, Yuho Sato, Takahiro Tachiki, Masami Hamada, Etsuko Kajita, Kuniyasu Kamiya, Kazuki Kaji, Koji Tsuda, Kumiko Ohara, Jong-Seong Moon, Jun Kitagawa, Masayuki Iki, the FORMEN study group

**Affiliations:** 1 Department of Public Health, Kindai University Faculty of Medicine, 377-2 Oono-higashi, Osaka-Sayama, Osaka, 589-8511 Japan; 2 Department of Hygiene and Public Health, Osaka Medical and Pharmaceutical University, 2-7 Daigakumachi, Takatsuki, Osaka, 569-8686 Japan; 3 Department of Hygiene and Public Health, Kansai Medical University, 2-5-1 Shin-machi, Hirakata, Osaka, 573-1010 Japan; 4 Department of Human Life, Jin-ai University, 3-1-1 Ootemachi, Echizen, Fukui, 915-8586 Japan; 5 Faculty of Nursing, Chukyo Gakuin University, 2216 Tokicho, Mizunami, Gifu, 509-6192 Japan; 6 Department of Rehabilitation, Kitasato University Kitasato Institute Hospital, Minato Ward, 5-9-1 Shirokane, Minato-ku, Tokyo, 108-8642 Japan; 7 Department of Nursing, Kio University, 4-2-2 Umami-naka, Koryo-cho, Nara, 635-0832 Japan; 8 Department of Rehabilitation Sciences, Graduate School of Medical Sciences, Kitasato University, 1-15-1 Kitasato, Sagamihara, Kanagawa 252-0373 Japan

**Keywords:** Bone mineral density, Bone-multiorgan crosstalk, Bone turnover marker, Community-dwelling Japanese elderly men, Death, Fracture, Osteoporosis, Quality of life, Risk model

## Abstract

**Background:**

The Fujiwara-kyo Osteoporosis Risk in Men (FORMEN) study was launched to investigate risk factors for osteoporotic fractures, interactions of osteoporosis with other non-communicable chronic diseases, and effects of fracture on QOL and mortality.

**Methods:**

FORMEN baseline study participants (in 2007 and 2008) included 2012 community-dwelling men (aged 65–93 years) in Nara prefecture, Japan. Clinical follow-up surveys were conducted 5 and 10 years after the baseline survey, and 1539 and 906 men completed them, respectively. Supplemental mail, telephone, and visit surveys were conducted with non-participants to obtain outcome information. Survival and fracture outcomes were determined for 2006 men, with 566 deaths identified and 1233 men remaining in the cohort at 10-year follow-up.

**Comments:**

The baseline survey covered a wide range of bone health-related indices including bone mineral density, trabecular microarchitecture assessment, vertebral imaging for detecting vertebral fractures, and biochemical markers of bone turnover, as well as comprehensive geriatric assessment items. Follow-up surveys were conducted to obtain outcomes including osteoporotic fracture, cardiovascular diseases, initiation of long-term care, and mortality. A complete list of publications relating to the FORMEN study can be found at https://www.med.kindai.ac.jp/pubheal/FORMEN/Publications.html.

## Background

In aging societies, osteoporotic fracture is a significant public health and economic burden not only for women but also for men. The incidence of hip fracture, the most serious type of osteoporotic fracture, is increasing and is projected to reach 13 million in 2050, with 31% of these (approximately 4 million) occurring in men [1]. It is well established that the incidence of hip fracture is higher in Caucasians than in Asians [2], but Asians will account for 45% of hip fractures globally in 2050 given the dramatic increase in elderly populations in Asia [1]. In Japan, nationwide surveys on the incidence of hip fracture have been conducted on six occasions since 1987 [3]. There were 176,000 estimated incident hip fractures in 2012 (triple the number from 1987), of which 22% occurred in men [3]. In addition, recent age-standardized incidence rates of hip fracture in men are increasing in Japan, while corresponding rates in women are stable [4]. This increasing trend highlights the need to establish a comprehensive strategy to prevent and manage osteoporosis in men, both on national and global levels.

Despite the substantial impact of osteoporosis on men, information on this topic is lacking, particularly in Asia. Only a few prospective cohort studies have been published on determinants of osteoporotic fracture in Hong Kong Chinese men [5] and Japanese men [6–8]. However, the sample size of these studies did not exceed 800, which is too low to meaningfully extract determinants [6–8]. Similarly, comprehensive geriatric assessments for evaluating risk factors of osteoporotic fracture are lacking. Such data are required to design rational clinical and preventive measures against osteoporosis and osteoporotic fractures. Against this backdrop, the Fujiwara-kyo Osteoporosis Risk in Men (FORMEN) study (Primary Investigator: Masayuki Iki), a large-scale community-based single-center prospective cohort study for elderly Japanese men, was conducted to address the following questions:

1. Which lifestyle and medical factors are associated with fracture risk in men?
2. Do bone turnover markers and other bone-related indices enhance the predictive value of bone density and other clinical risk factors for fracture risk in men?
3. To what extent do fractures increase the risk of being dependent on care provided from long-term care insurance (LCI) and affect quality of life (QOL) in men?
4. Do existing vertebral fractures and incident osteoporotic fractures increase the risk of death in men?
5. Are osteoporosis and osteoporotic fractures associated with the incidence or progression of non-communicable diseases including cardiovascular diseases (CVD) and diabetes mellitus?

## Methods/design

### Study setting and participants

The FORMEN study is an ancillary study of a larger prospective cohort study, the Cohort Study for Functioning Capacity and Quality of Life in Elderly Japanese (Primary Investigator: Norio Kurumatani), referred to as the “Fujiwara-kyo study” after the study area where the first capital of Japan (Fujiwara-kyo) was established in 694 AD. The Fujiwara-kyo study aims to provide a scientific basis for a comprehensive strategy to prevent frailty, increase the number of healthy life years, and promote the functioning capacity and quality of life of elderly men and women in Japan.

To achieve these aims, participants should be representative of elderly men and women who live independently in a community and can participate in various preventive activities conducted by their municipalities. Thus, inclusion criteria for participants of the Fujiwara-kyo study are as follows:

1. Aged 65 years or older at baseline,
2. Living in their homes in the cities of Kashihara, Nara, Yamato-Kooriyama, and Kashiba,
3. Able to walk without the assistance of another person,
4. Able to provide self-reported information, and
5. Able to understand and provide written informed consent.

The FORMEN study focuses on bone health of male participants of the Fujiwara-kyo study. Most non-skeletal measurements and interviews were conducted in the Fujiwara-kyo study and form a comprehensive geriatric and gerontologic basis for the FORMEN study to evaluate risk factors of osteoporosis and osteoporotic fractures in elderly men.

The sample size necessary for the FORMEN study was set at 2000. This number is needed to obtain significant results from logistic regression analysis with a two-tailed level of significance of 5% and a statistical power of 80%, when a risk factor for fracture is assumed to exist in 20% of participants of the FORMEN baseline study and to increase a background fracture risk of 1% per year by 75% during the 5-year follow-up period.

Written informed consent was obtained from each subject before participating in the study. The protocol of the FORMEN study was approved by the Ethics Committee of Kindai University Faculty of Medicine (Approval number 19-32, 29-162).

### Study design and timeline

Baseline clinical surveys of the Fujiwara-kyo study were conducted in 2007 and 2008. Clinical follow-up surveys, which included similar study items as the baseline survey as well as items identifying outcomes, were conducted in 2012–2013 and 2017–2019. Supplemental mail and telephone surveys and a visit survey were conducted for those who did not participate in the clinical surveys in order to identify the incidence of outcomes.

### Measurements

The baseline survey of the FORMEN study covered a wide range of bone health-related indices such as BMD measurements, trabecular microarchitecture assessment, X-ray absorptiometric imaging of the spine for detecting vertebral fractures, and biochemical markers of bone turnover, as well as comprehensive geriatric assessment items obtained in the Fujiwara-kyo study. Follow-up surveys were designed to identify outcomes such as the incidence of vertebral fractures and clinical fractures, change in BMD, LCI benefits, and death. Measurements made in the FORMEN study are shown in Table 1.

**Table 1:** Study measurements conducted in each phase of the FORMEN study

Phase	Measurements
Baseline surveys in 2007–2008	Bone mineral density measurements at the spine and hip by DXA
	Vertebral fracture assessment by SXA
	Trabecular bone microarchitecture assessment at the spine by TBS
	Fasting blood samples taken: fasting glucose, HbA1c, insulin, lipids, bone turnover markers (OC, TRACP5b, P1NP, and CTX), ucOC, pentosidine, esRAGE, and high sensitivity CRP assayed; and serum aliquots stored at –80°C
	Interviews to obtain information on past history of illness, lifestyle factors, socioeconomic status, health-related QOL (SF-36), geriatric depression scale, and ADL and IADL scales
	Mini-mental state examination
	Weight, height, waist circumference, and blood pressure measurements
	Physical performance tests (grip strength, 10-m gait speed, one-foot standing balance with eyes open, and knee flexor and extensor strength)
Mail and telephone surveys in 2008 and 2009	Questionnaire and interviews to obtain information on incidence of outcomes (death, LCI benefits, fractures, and CVD)
Clinical follow-up surveys in 2012–2013	Bone mineral density measurements at the spine and hip by DXA
	Vertebral fracture assessment by SXA
	Fasting blood samples taken: fasting glucose, HbA1c, insulin, lipids, osteocalcin, ucOC, and high-sensitivity CRP assayed; and serum aliquots stored at –80°C
	Interviews to obtain information on incidence of fractures, major diseases and treatments, health-related QOL (SF-36), geriatric depression scale, and ADL and IADL scales
	Mini-mental state examination
	Weight, height, waist circumference, and blood pressure measurements
	Ultrasonography at the carotid artery
	Physical performance tests (grip strength, 10-m gait speed, one-foot standing balance with eyes open, and five times sit to stand test)
Supplemental mail or visit survey in 2013–2014	Questionnaire and interviews to obtain information on incidence of outcomes (death, LCI benefits, fractures, and CVD)
Clinical follow-up surveys in 2017–2019	Bone mineral density measurements at the spine and hip by DXA
	Vertebral fracture assessment by SXA
	Body composition measurements by DXA
	Fasting blood samples taken: fasting glucose, HbA1c, insulin, lipids, osteocalcin, ucOC, and high-sensitivity CRP assayed; and serum aliquots stored at –80°C
	Interviews to obtain information on incidence of fractures, major diseases and treatments, and health-related QOL (SF-36)
	Mini-mental state examination
	Weight, height, waist circumference, and blood pressure measurements
	Ultrasonography at the carotid artery and baPWV measurement
	Physical performance tests (grip strength, 10-m gait speed, one-foot standing balance with eyes open, and five times sit to stand test)
Supplemental mail and telephone surveys in 2019	Questionnaire and interviews to obtain information on incidence of outcomes (death, LCI benefits, fractures, and CVD)
Ongoing	Inquiry to municipal office regarding LCI benefits, death, and moving out of study area

*DXA* dual-energy X-ray absorptiometry, *SXA* single-energy X-ray absorptiometry, *TBS* trabecular bone score, *OC* osteocalcin, *TRACP5b* tartrate-resistant acid phosphatase isoenzyme 5b, *P1NP* type 1 procollagen N-terminal propeptide, *CTX* type 1 collagen C-terminal telopeptide, *ucOC* undercarboxylated OC, *esRAGE* endogenous secretory receptor for advanced glycation end-product, *CRP* c-reactive protein, *QOL* quality of life, *ADL* activity of daily living, *IADL* instrumental ADL, *LCI* long-term care insurance, *CVD* cardiovascular disease

### Assessment of predictors

#### Bone mass measurements

L1 to L4 vertebrae and the proximal femur were scanned in posteroanterior projection with a dual-energy X-ray absorptiometry (DXA) scanner (QDR4500A, Hologic Inc., Bedford, MA, USA) installed in a mobile test room. BMD of the spine and femoral neck, trochanteric, intertrochanteric, and Ward’s triangle regions were obtained [9].

#### Prevalent vertebral deformity assessment

Thoracolumbar vertebrae were imaged by single-energy X-ray absorptiometry (SXA) (T4 through L4) at baseline and each of the follow-up surveys by certified radiological technologists using the same scanner that was used to measure BMD. Vertebral deformities were diagnosed at baseline according to Genant’s semiquantitative assessment method [10].

#### Trabecular bone microarchitecture assessment

Spine DXA images archived at baseline were further analyzed by the TBS iNsight software (Version 2.1, Med-Imaps, Bordeaux, France) to obtain the trabecular bone score (TBS). TBS is a texture parameter that quantifies local variations in gray level distribution of DXA images. TBS is not a direct physical measure of bone microarchitecture but is highly correlated with its three-dimensional parameters [11, 12].

#### Bone turnover markers and other laboratory measurements

In each survey of the FORMEN study, venous blood samples were drawn from all participants on their visits from 09:00 to 14:00 after at least a 7-h fast. Serum samples were stored at −80°C until use.

As markers of bone formation, intact molecule of osteocalcin (OC) was measured by a two-site immunoradiometric assay (IRMA) (BGP IRMA kit; Mitsubishi Kagaku Iatron, Tokyo, Japan), and type I procollagen N-terminal propeptide by a radioimmunoassay (Procollagen Intact OINO; Orion Diagnostica, Espoo, Finland). As bone resorption markers, tartrate-resistant acid phosphatase isoenzyme 5b was measured by a fragment-absorbed immunocapture enzyme assay (Osteolinks-TRAP-5b; Nitto Boseki, Kooriyama, Japan), and type I collagen C-terminal telopeptide by enzyme-linked immunosorbent assay (ELISA) kits (Serum CrossLaps; Immunodiagnostic Systems Ltd., Boldon, UK).

#### Other bone-related measurements

Serum undercarboxylated OC (ucOC) was measured as a marker for vitamin K sufficiency by an electrochemiluminescence immunoassay (Picolumi ucOC; Sanko Junyaku Co. Ltd., Tokyo, Japan), serum pentosidine levels by competitive ELISA kits (FSK PEN ELISA kit; Fushimi Pharmaceutical Co., Marugame, Japan), endogenous secretory receptor for advanced glycation end products (esRAGE) by ELISA kits (B-Bridge esRAGE ELISA kit; B-Bridge International, Mountain View, CA, USA), and high-sensitivity C-reactive protein levels by nephelometry (N-Latex CRP II; Siemens Healthcare Diagnostics Inc., Deerfield, IL, USA).

### Assessment of outcomes

#### Incidence of morphometric vertebral fractures

An incident vertebral fracture was diagnosed morphometrically based on SXA images of the spine when the anterior, central, or posterior height of a vertebra are reduced by 20% or more on follow-up images compared to baseline and when the vertebra also satisfies the definition of grade 2 or 3 fracture by Genant’s method [10].

#### Incidence of clinical fractures

The time of a fracture event, the skeletal site of fracture, the situation in which the fracture occurred, and use of radiographs for forming the diagnosis of fracture by a physician were obtained during interviews at each follow-up survey. Supplemental mail and telephone surveys were conducted just after the follow-up surveys to obtain the same information from non-participants. Fragility fracture was defined as a fracture that occurred without strong external force, caused pain, and was diagnosed by a physician with radiographic examination.

The above method was validated using 21 incident fractures identified during the first 2 years of follow-up. Of these, 19 participants provided consent to contact the attending surgeon in order to confirm the occurrence, date, and skeletal site of fractures. All surgeons responded to our inquiries and confirmed the occurrence of all self-reported fractures and that differences between the self-reported date and the real date of fracture events were within 6 months. Self-reported fracture sites seldom differed from the correct sites. We concluded that the false-positive rate for fracture detection using the present method was extremely low and that errors in fracture dates and skeletal sites would be within an acceptable range.

#### Ultrasonography at the carotid artery and pulse wave velocity measurement

Ultrasonographic examination of carotid arteries was performed with participants in the supine position using high-resolution B-mode ultrasonography with a 7.5 MHz linear probe (SSA-660A Xrio, Thoshiba Medical Systems Inc., Tokyo, Japan). Intima-media thickness of the far wall at the carotid bifurcation (BIF-IMT) was measured in four views, i.e., longitudinal and transverse sections of the bifurcation of the right and left arteries, and the maximum value of the four wall measurements was adopted as the BIF-IMT value according to the Guidelines for Ultrasonic Assessment of Carotid Artery Disease by the Japan Academy of Neurosonology [13].

Brachial-ankle pulse wave velocity (baPWV) was measured using a volume plethysmographic apparatus (Form PWV/ABI; Fukuda Colin Co., Ltd. Tokyo, Japan). Participants were examined while resting in a supine position with the measurement device set to record baPWV. Both the brachium and ankle were wrapped with cuffs connected to plethysmographic sensors in order to determine brachial and post-tibial arterial pressure waveforms. baPWV was calculated as the difference (cm) between the path lengths from the heart to the brachium and from the heart to the ankle divided by the traveling time delay (s) of the pulse wave detected at the ankle compared with that at the brachium.

#### Identification of deaths

Participant deaths that occurred from baseline to the end of March 2019 were identified through supplemental mail and telephone surveys for non-participants in the clinical follow-up surveys and inquiries to the municipal resident registry for non-respondents to the supplemental surveys. We obtained the dates but not the causes of deaths since the cause of death was not included in the resident registry.

## Key findings

**Fig. 1:**
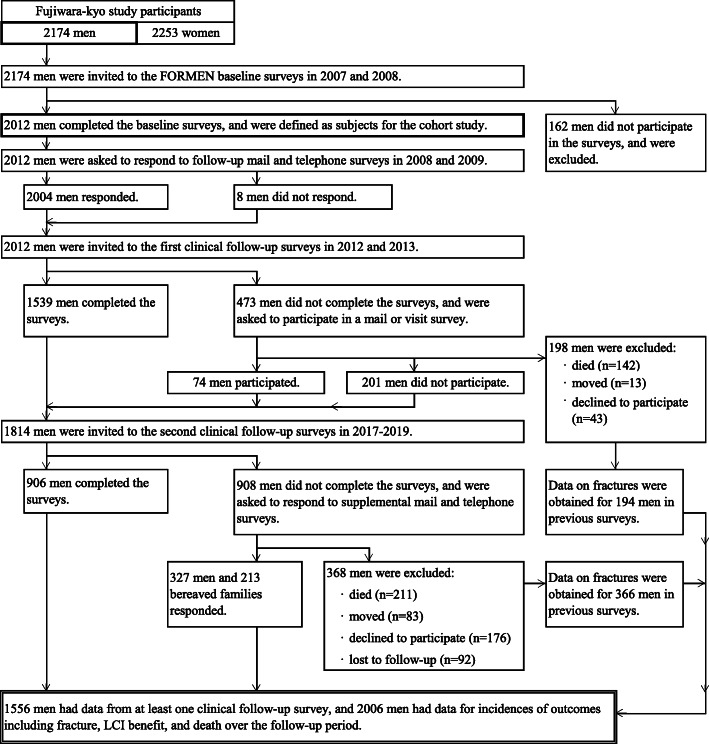
Flow diagram of participant selection in the FORMEN study

A flow diagram of participant selection is shown in Fig. 1. Of the 4427 participants of the Fujiwara-kyo study, 2174 were men and were invited to participate in the FORMEN study. Of these, 2012 completed the baseline survey. Among them, 1556 men (77.3%) participated in at least one clinical follow-up survey, and 450 additional men responded to the supplemental surveys. Consequently, we identified 220 participants with incident fractures and 566 deaths from baseline to the end of March 2019.

Table 2 shows a comparison of baseline characteristics between participants and non-participants of the clinical follow-up surveys. Non-participants were significantly older; shorter in height; lighter in weight; consumed less energy, less calcium, and less vitamin K; had lower bone mineral density (BMD) at the hip and femoral neck; and had higher values of glycemic indices, cystatin-c, and c-reactive protein than participants. More non-participants were current smokers than participants, while fewer non-participants were habitual drinkers than participants. History or present involvement of type 2 diabetes mellitus (T2DM) was significantly higher in non-participants than participants. Although clinical data for non-participants of the follow-up surveys were lacking, outcome data including clinical fractures, LCT benefits, and deaths were available for a total of 2006 men (99.7% of all participants at baseline).

**Table 2:** Comparison of baseline characteristics between participants and non-participants of clinical follow-up surveys

			Clinical follow-up surveys				
	Participants of baseline survey		Participants		Non-participants		*p*-value for difference
	*N*	Average value	*N*	Average value	*N*	Average value	
Baseline characteristics							
Age (years)	2012	73.0 ± 5.2	1556	72.4±4.9	456	75.1±5.8	<0.001
Height (cm)	2012	162.8 ± 5.7	1556	163.0±5.8	456	162.3±5.5	0.026
Weight (kg)	2012	60.9 ± 8.6	1556	61.2±8.4	456	59.8±9.1	0.004
BMI (kg/m^2^)	2012	22.9 ± 2.8	1556	23.0±2.7	456	22.7±3.1	0.042
Waist circumference (cm)	2012	85.5 ± 7.9	1552	85.6±7.7	460	85.3±8.7	0.452
Physical activity (Mets·min/day)	1848	181×/÷2.7	1443	184×/÷2.8	405	169×/÷2.6	0.129
Energy intake (kcal/day)	1689	2047±354	1338	2067±348	351	1971±367	<0.001
Calcium intake (mg/day)	1689	514±190	1338	523±190	351	479±185	<0.001
Vitamin D intake (mg/day)	1689	9.7±6.0	1338	9.6±6.0	351	10.2±6.1	0.099
Vitamin K intake (mg/day)	1689	207±111	1338	212±111	351	187±109	<0.001
LS-BMD (g/cm^2^)	1914	1.026±0.191	1493	1.028±0.189	421	1.018±0.198	0.347
TH-BMD (g/cm^2^)	2006	0.878±0.126	1552	0.885±0.125	454	0.857±0.128	<0.001
FN-BMD (g/cm^2^)	2006	0.740±0.115	1552	0.746±0.114	454	0.721±0.117	<0.001
PTH (pg/ml)	1828	20.4×/÷1.6	1427	20.4×/÷1.5	401	20.2×/÷1.6	0.678
OC (ng/ml)	1963	4.90×/÷1.5	1519	4.92×/÷1.5	444	4.82×/÷1.5	0.334
P1NP (mg/l)	1417	34.7×/÷1.5	1119	34.5×/÷1.5	298	35.4×/÷1.5	0.359
TRACP5b (mU/dl)	2003	212.3×/÷1.7	1549	209.2×/÷1.7	454	223.1×/÷1.8	0.031
CTX (ng/ml)	1545	0.205×/÷1.6	1213	0.20×/÷1.6	332	0.21×/÷1.7	0.315
ucOC (ng/ml)	1970	2.89×/÷1.9	1526	2.87×/÷1.8	444	2.95×/÷2.0	0.487
FPG (mg/dl)	2008	104.2±31.4	1553	102.6±27.1	455	109.6±42.5	<0.001
HbA1c (%)	2010	5.7±0.8	1555	5.7±0.7	455	5.8±0.9	0.002
Insulin (mU/l) ^a^	1957	4.9×/÷2.1	1513	4.8×/÷2.0	444	5.3×/÷2.3	0.036
Homa-IR ^b^	1810	1.1×/÷2.0	1411	1.1×/÷2.0	399	1.1×/÷2.1	0.328
Albumin (mg/dl)	2012	4.5±0.3	1556	4.6±0.3	456	4.5±0.3	<0.001
Total protein (mg/dl)	2012	7.3±0.5	1556	7.3±0.5	456	7.3±0.5	0.927
AST (mg/dl)	2012	25.8×/÷1.4	1556	25.5×/÷1.3	456	26.9×/÷1.4	0.002
ALT (mg/dl)	2012	20.1×/÷1.6	1556	20.2×/÷1.5	456	19.9×/÷1.6	0.627
Triglyceride (mg/dl)	2012	114.3×/÷1.6	1556	115.3×/÷1.6	456	110.8×/÷1.6	0.117
LDL (mg/dl)	2012	118.9×/÷1.3	1556	120.2×/÷1.3	456	114.7×/÷1.3	<0.001
HDL (mg/dl)	2012	53.8×/÷1.3	1556	54.1×/÷1.3	456	53.0×/÷1.3	0.169
Creatinine (mg/dl)	2012	0.87×/÷1.2	1556	0.87×/÷1.2	456	0.89×/÷1.3	0.134
eGFR (mL/min/1.73 m^2^)	2012	65.6×/÷1.2	1556	66.1×/÷1.2	456	64.2×/÷1.3	0.022
Cystatin-C (mg/l)	1834	0.85×/÷1.2	1433	0.83×/÷1.2	401	0.89×/÷1.3	<0.001
hsCRP (ng/ml)	2006	648.0×/÷3.2	1553	621.6×/÷3.2	453	747.1×/÷3.2	0.003
Uric acid (mg/dl)	2012	5.7×/÷1.3	1556	5.7×/÷1.3	456	5.7×/÷1.3	0.474
History or present situation (*N*, %)							
Current smoker	2012	340, 16.9%	1556	240, 15.4%	456	100, 22.0%	0.001
Habitual drinker	2012	956, 47.5%	1556	775, 49.8%	456	181, 39.8%	<0.001
Gastrectomy	2012	136, 6.8%	1556	103, 6.6%	456	33, 7.3%	0.714
Prostate cancer	2012	32, 1.6%	1556	23, 1.5%	456	9, 2.0%	0.592
Asthma	2012	12, 0.6%	1556	10, 0.6%	456	2, 0.4%	0.882
Connective tissue diseases	2012	15, 0.7%	1556	12, 0.8%	456	3, 0.7%	0.805
Glucocorticoid therapy	2012	27, 1.3%	1556	22, 1.4%	456	5, 1.1%	0.778
Hyperthyroidism	2012	1, 0.05%	1556	1, 0.06%	456	0, 0%	1.000
Parathyroid diseases	2012	1, 0.05%	1556	1, 0.06%	456	0, 0%	1.000
Type 1 diabetes mellitus	2012	4, 0.2%	1556	4, 0.3%	456	0, 0%	0.628
Type 2 diabetes mellitus	2012	364, 18.1%	1556	266, 17.1%	456	98, 21.5%	0.036

Data are expressed as arithmetic mean ± SD or geometric mean ×/÷ SD

*N* number of participants, *BMI* body mass index, *BMD* bone mineral density, *LS* lumbar spine (L1–L4), *TH* total hip, *FN* femoral neck, *PTH* parathyroid hormone, *OC* osteocalcin, *P1NP* procollagen type 1 N-terminal propeptide, *TRACP5b* tartrate-resistant acid phosphatase isoenzyme 5b, *CTX* type 1 collagen C-terminal telopeptide, *ucOC* undercarboxylated OC, *FPG* fasting plasma glucose, *HbA1c* glycated hemoglobin A1c, *Homa-IR* homeostasis model assessment-insulin resistance, *AST* aspartate aminotransferase, *ALT* alanine aminotransferase, *LDL* low-density lipoprotein cholesterol, *HDL* high-density lipoprotein cholesterol, *eGFR* estimated glomerular filtration rate, *hsCRP* high-sensitivity C-reactive protein

^a^For 1957 participants without current insulin treatment

^b^For 1810 participants without current insulin treatment and with FPG <140 mg/dl

We explored risk factors for low BMD by cross-sectional analyses using data from the FORMEN baseline study. Patients with end-stage kidney disease or predialysis renal failure have been reported to exhibit decreased BMD [14, 15]. However, we found that higher serum creatinine levels were associated with higher BMD at the femoral neck in participants of the FORMEN study, while cystatin-c levels were not [16]. Creatinine may not be an appropriate index to evaluate the renal effect on BMD in people with normal to moderate impairment of renal function, since greater muscle mass results in higher levels of serum creatinine and BMD. With regard to lifestyle factors, we found that current smoking [17], habitual drinkers with an ethanol intake of more than 60 g/day [18], no intake of fermented soybean product (natto) [19], and low intake of vitamin K [19] and milk [20] were significantly associated with low BMD.

We also explored risk factors for fracture. Patients with T2DM reportedly have increased BMD but an elevated risk of fracture [21]. A similar result was observed in the FORMEN study [22], but fracture risk in patients with T2DM was greater and appeared at an earlier stage of T2DM than reported in a meta-analysis of Caucasian populations [21], suggesting a potential ethnic difference in susceptibility of bone strength to hyperglycemia. One of the causes of increased fracture risk in T2DM patients may be an increase in retention of advanced glycated end-product (AGE) in bone tissue [23]. We found that esRAGE, a decoy receptor for AGE, was associated with a lower risk of osteoporotic fracture, while pentosidine, an AGE, was associated with a higher risk [24]. In addition, patients with T2DM had a lower TBS than those without [25]. Thus, T2DM may increase fracture risk through the retention of AGE and deterioration of trabecular bone microarchitecture.

Gastric cancer is one of the most common cancers in Japan, and 70% of patients with gastric cancer are indicated for gastrectomy. FORMEN participants with a history of gastrectomy showed a three-fold higher risk of osteoporotic fracture than those without [26]. This increase in risk persisted for more than 20 years after gastrectomy, regardless of the reason for undergoing the procedure. We found that participants with lower serum uric acid levels also had an increased risk of vertebral fracture than those with higher levels [27]. This suggests that excessive treatment of patients with gout by uric acid-lowering medications may increase the risk of vertebral fracture. FRAX®, a fracture risk assessment tool, is increasingly being used in clinical settings, but its predictive ability is not high [28]. In the FORMEN study, a lower TBS was related to a higher risk of major osteoporotic fracture independently of the risk predicted by FRAX® score [29], suggesting that TBS may improve the predictive ability of FRAX® for fracture risk [30].

OC, a biochemical marker for bone formation, is a hormone that regulates energy and glucose metabolism in mice, and its active form is ucOC [31]. Serum ucOC levels were inversely associated with glycemic indices, and patients with T2DM showed significantly lower ucOC levels than those without [32]. These findings suggest that ucOC may function in a similar manner in humans as in mice.

Patients with hip fracture and clinically manifested vertebral fracture have an increased risk of mortality [33], but it is unclear whether fractures increase the risk of death via frailty, which is known to increase the risk of fracture and death. In the FORMEN study, participants with incident fractures showed a three-fold higher risk of death than those without fracture, and this increased risk was independent of frailty indices, including physical performance test results [34].

A complete list of publications relating to the FORMEN study can be found at https://www.med.kindai.ac.jp/pubheal/FORMEN/Publications.html.

## Discussion

The FORMEN study has some strengths over previous studies. First, the sample size of the FORMEN study is the largest of this kind in Japan. Thus, risk factors of vertebral and osteoporotic fractures can be evaluated with robust statistical power. Second, the FORMEN study comprehensively covered skeletal measures relevant to osteoporosis, including vertebral fracture assessment and TBS, as well as conventional BMD measurements. Third, biochemical markers of bone turnover in sera were measured for all participants, with remaining sera stored at −80°C for measurements of new markers. Fourth, the FORMEN study comprehensively evaluated potential risk factors of osteoporosis and osteoporotic fractures based on geriatric and gerontologic assessments. Finally, there is no inter-center variation due to the single-center study design.

The FORMEN study also has potential limitations worth noting. First, participants were not randomly selected. Thus, caution must be exercised when generalizing the results. Second, although large, the sample size of the FORMEN study may still be insufficient to assess risk factors for fracture with relatively low risk or low prevalence. Third, BMD measurements did not include whole body BMD or volumetric BMD obtained by quantitative computed tomography. Fourth, vertebral deformity detected with SXA images might underestimate the prevalence of vertebral fractures compared to diagnosis with conventional radiographs since concave type or endplate fractures may have been missed [35]. Fifth, we did not cross-check all self-reported fracture events with data from medical records, although our validation study and previous studies indicated that self-reported data are relatively accurate [36, 37]. Finally, a survey of causes of death is not included in the study protocol and thus was not performed.

In conclusion, the prospective cohort data from the FORMEN study will provide reliable evidence regarding the risk factors of osteoporosis and osteoporotic fractures and establish a comprehensive strategy to promote bone health in men.

## Data Availability

The FORMEN dataset is not freely available, but the Study Group has collaborated with several other groups to share study data and encourages new collaborations. Potential collaborators are invited to contact the Secretary General (Yuki Fujita, PhD, pbl-h@med.kindai.ac.jp) at the administrative office of the FORMEN Study Group at the Department of Public Health, Kindai University Faculty of Medicine, Osaka-Sayama, Osaka, Japan.

## Abbreviations

AGE: Advanced glycated end-product
BIF: Carotid bifurcation
baPWV: Brachial-ankle pulse wave velocity
BMD: Bone mineral density
CVD: Cardiovascular diseases
DXA: Dual-energy X-ray absorptiometry
ELISA: Enzyme-linked immunosorbent assay
esRAGE: Endogenous secretory receptor for advanced glycation end products
FORMEN: Fujiwara-kyo Osteoporosis Risk in Men
IRMA: Immunoradiometric assay
IMT: Intima-media thickness
LCI: Long-term care insurance
OC: Osteocalcin
QOL: Quality of life
SXA: Single-energy X-ray absorptiometry
T2DM: Type 2 diabetes mellitus
TBS: Trabecular bone score
ucOC: Undercarboxylated osteocalcin
